# Safety and efficacy of two-port thoracoscopic aortic valve replacement

**DOI:** 10.1186/s13019-022-02086-0

**Published:** 2023-01-07

**Authors:** Tong Tan, Peijian Wei, Yanjun Liu, Huanlei Huang, Jian Zhuang, Jimei Chen, Jian Liu, Huiming Guo

**Affiliations:** 1 Shantou University Medical College, Shantou, China; 2 Department of Cardiovascular Surgery, Guangdong Cardiovascular Institute, Guangdong Provincial People’s Hospital (Guangdong Academy of Medical Sciences), Southern Medical University, Guangzhou, China; 3 Guangdong Provincial Key Laboratory of South China Structural Heart Disease, Guangzhou, China; 4 Department of Structure Heart Center, Fuwai Hospital, National Center for Cardiovascular Diseases, Chinese Academy of Medical Sciences and Peking Union Medical College, Beijing, China

**Keywords:** Minimally invasive, Endoscopic surgery, Totally thoracoscopic aortic valve replacement, Aortic valve replacement, Acute kidney injury

## Abstract

**Background:**

Pure aortic valve disease is common and has been treated with sternotomy aortic valve replacement for decades. Minimally invasive cardiac surgery has been widely used in atrioventricular valve lesions, but totally thoracoscopic aortic valve replacement has rarely been reported.

**Method:**

The profiles of 9 patients who were diagnosed with severe aortic valve diseases and treated with two-port thoracoscopic aortic valve replacement between February 2021 and February 2022 were retrospectively reviewed. The clinical data, including baseline characteristics, operative data, postoperative complications, and short-term outcomes, were reported.

**Results:**

All nine patients successfully underwent two-port thoracoscopic aortic valve replacement, with a cardiopulmonary bypass time of 137.56 ± 27.99 min and an aortic cross-clamp time of 95.33 ± 17.96 min. Seven (77.78%) patients underwent mechanical valve replacement, and two (22.22%) patients underwent bioprosthetic valve replacement. Two (22.22%) patients underwent a concomitant aortic root enlargement procedure. There were no intraoperative or postoperative deaths. The incidence of procedural complications was 0%, while the results of ventilation time, intensive care unit stay length, blood transfusion, chest tube drainage, and kidney function were satisfactory.

**Conclusion:**

Two-port thoracoscopic aortic valve replacement is a safe and effective surgical treatment option for carefully selected patients with pure aortic valve diseases.

**Supplementary Information:**

The online version contains supplementary material available at 10.1186/s13019-022-02086-0.

## Background

The prevalence of aortic valve disease presenting as aortic regurgitation (AR) or aortic stenosis (AS) increases with age [1–3]. Once severe clinical conditions such as dyspnea and syncope develop and remain poorly controlled by medicine, surgery becomes the standard therapy for these patients. Sternotomy aortic valve replacement (SAVR) has been performed for more than 50 years as a safe and feasible procedure. SAVR provides excellent exposure and is convenient for establishing cardiopulmonary bypass (CPB). However, full sternotomy also has several disadvantages, such as extensive trauma with prolonged recovery, increased blood loss, and apparent postoperative scarring. Minimally invasive endoscopic surgery, an alternative to SAVR that also reduces surgical trauma and speeds up recovery, has rapidly developed in recent years. Cosgrove first described a minimally surgical aortic valve replacement (Mini-SAVR) through a right parasternal approach [4]. Since then, many subtypes of Mini-SAVR have been explored, including a midline incision with J-shape sternotomy at the third to fourth intercostal space; a right anterolateral incision at the second intercostal space; and “T-shape”, “I-shape” or “Y-shape” variations of partial sternotomy [5]. Along with the technique of video-assisted thoracic surgery (VATS), the combination of thoracoscopy and Mini-SAVR helps surgeons make smaller incisions but obtain larger fields of view. Totally thoracoscopic aortic valve replacement is one of the less invasive and complementary techniques for Mini-SAVR, but its reports have been limited. In this study, we describe the preliminary results and our experience with two-port thoracoscopic aortic valve replacement (TTAVR). Our aim was to evaluate the safety and efficacy of TTAVR.

## Methods

### Study population and data collection

The clinical profiles of 9 consecutive patients who underwent TTAVR by a single operator between February 2021 and February 2022 were retrospectively analyzed. Patients were considered candidates for TTAVR if the following parameters were met: (1) pure aortic valve diseases, including bicuspid valve disease, rheumatic valve disease, and severe aortic regurgitation that required surgical treatment according to current recommendations [6]; (2) adult patients aged between 18 and 70 years whose Society of Thoracic Surgeons (STS) scores were < 4%; and (3) patients selected minimally invasive surgery following a full explanation of the procedures. The exclusion criteria were aortic valve disease combined with other valve diseases, coronary artery disease requiring simultaneous surgery, severe pleural adhesions from a previous history of right thoracic surgery or constrictive pericarditis, and severe peripheral vascular disease limiting the establishment of CPB. The clinical data, including age, sex, body mass index (BMI), comorbidities, New York Heart Association (NYHA) functional class, and personal history, were collected to calculate STS scores. Laboratory findings, operation records, and in-hospital events were extracted from the electronic medical records. The primary endpoints were procedure success rate, in-hospital mortality, and the occurrence of adverse effects, including reoperation for bleeding, stroke, renal failure, new-onset atrial fibrillation and third-degree atrial ventricular block.

### Surgical procedures

After the administration of general anesthesia, the patient was ventilated with a double-lumen endotracheal tube and was positioned in a supine position with a pillow or an airbag beneath the right scapula to elevate the right hemithorax at 30°. Transesophageal echocardiography (TEE) was routinely used to assess aortic valve lesions and cardiac function before the beginning of surgery. For the drainage of blood, the superior vena cava was cannulated through the right jugular vein. A 2–3 cm transverse incision in the right groin was made to isolate the femoral artery and vein. After systemic heparinization, CPB was performed with femoral arterial (17–20F) and venous (24–28F) cannulation.

**Fig. 1:**
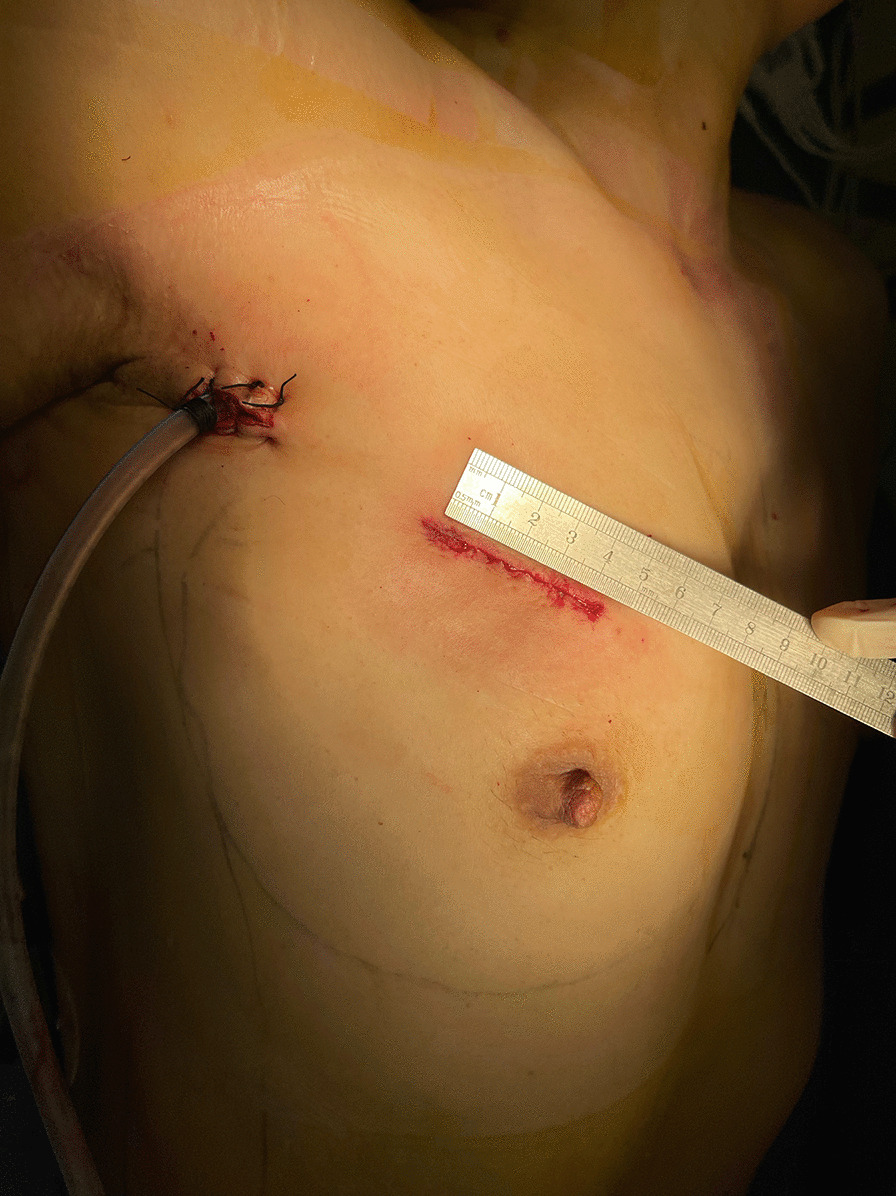
Two-port incisions for totally thoracoscopic aortic valve replacement

**Fig. 2:**
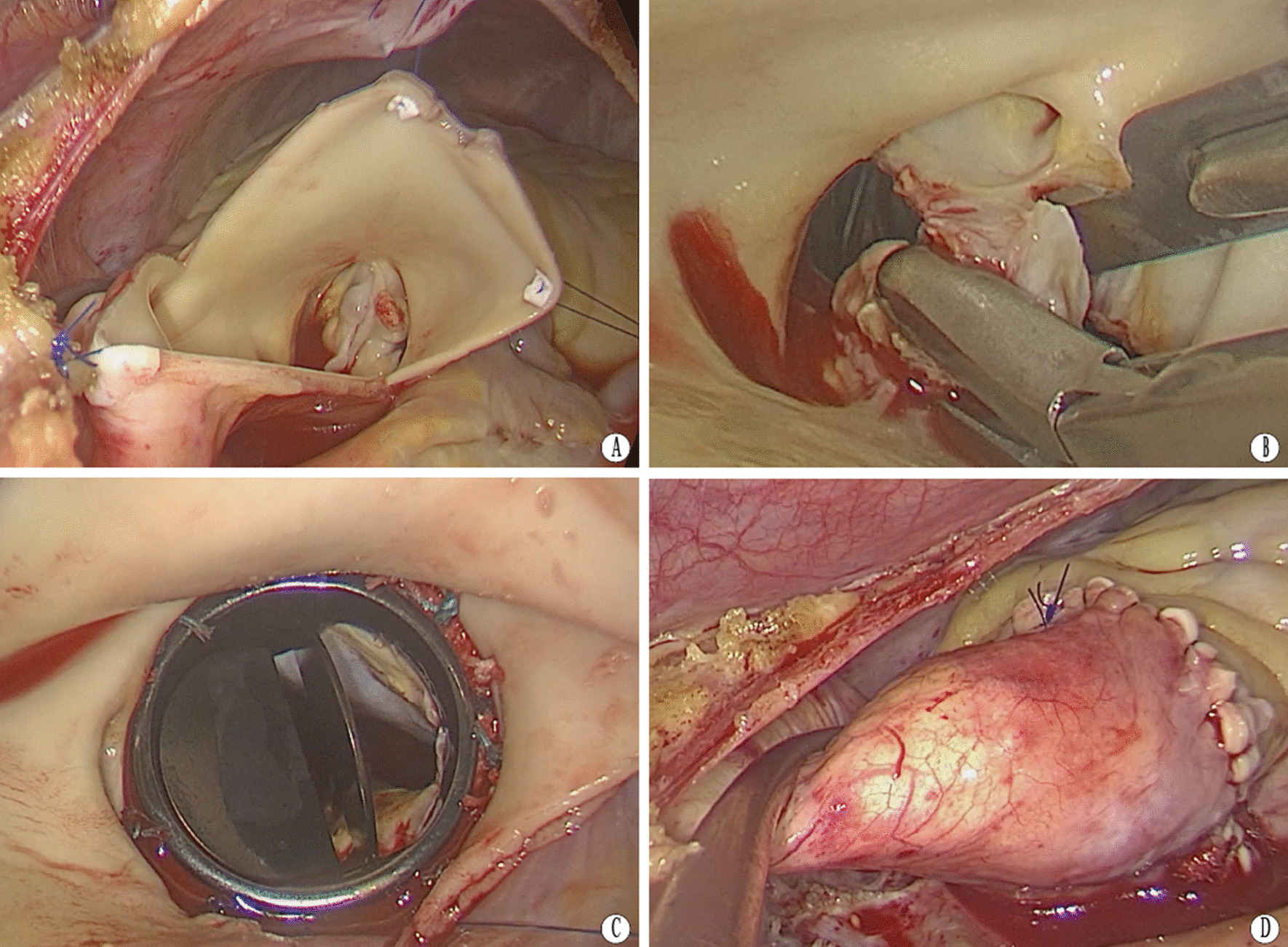
Intraoperative views in two-port thoracoscopic aortic valve replacement. **A** The aorta was dragged outward to obtain good exposure of the aortic valve; **B** The aortic valve was completely resected; **C** The prosthetic valve was settled; **D** The aortic incision was closed

The working port (3–4 cm) was placed at the third intercostal space on the mid-clavicular line, while the thoracoscopic port was placed in a 1.5 cm incision at the third intercostal space at the level of the anterior axillary line. A soft tissue retractor was inserted to create a space for two surgical instruments in the working port. The retractor might lead to pain postoperatively, but more importantly, care must be taken not to damage the internal mammary arteries and thus avoid bleeding. The thoracoscopic port was used to place the thoracoscope, the left ventricle vent catheter, the transthoracic Chitwood clamp during surgery, and the drainage tube after surgery (Fig. 1). Parallel to the right phrenic nerve, the pericardium was opened and suspended. After applying mild systemic cooling, a transverse incision was made through the aorta, and then the aorta was dragged outward by three sutures with Dacron felt patches (Fig. 2A). The cold blood cardioplegia solution was perfused through the left and right coronary arteries for myocardial protection. Next, the aortic valve lesion was probed, and the aortic valve was resected from the annulus (Fig. 2B). Any calcification was debrided by a rongeur. A mechanical valve or a bioprosthetic valve was selected. For an artificial valve that could not be compressed and transferred through the working port, a tissue distractor is essential to enlarge the port temporarily. The size of the artificial valve depends on the patient’s body surface area and the size of the aortic annulus. The corresponding valve measurement should be able to advance through the aortic annulus without a significant gap or resistance. If the size of the annulus is between two consecutive valve sizes, we prefer the larger size with an aortic root enlargement procedure (Additional file 1: Video 1). The artificial valve was settled using 2–0 sutures with Dacron felt patches (Fig. 2C). Finally, the incision was closed using 4–0 Prolene sutures (Fig. 2D). TEE was performed to confirm the function of the aortic valve following de-airing and Chitwood clamp removal.

### Statistical analysis

Statistical analysis was performed using SPSS software (IBM SPSS Statistics for Mac, Version 26.0). Continuous data are presented as the mean ± standard deviation (SD) for normally distributed variables and as the median with interquartile range (IQR) for nonnormally distributed variables. Categorical data are presented as the frequency and percentage.

## Results

Table 1 summarizes the baseline characteristics of the patients. Among nine patients who underwent TTAVR between February 2021 and February 2022, six (66.67%) were male. The mean age was 50.89 ± 5.30 years, and the mean BMI was 23.13 ± 2.09 kg/m^2^. Five (55.56%) patients belonged to NYHA class II, and four (44.44%) belonged to NYHA class III. Five (55.56%) patients were diagnosed with severe AS, and four (44.44%) patients had severe AR. Six (66.67%) patients had bicuspid aortic valve morphology. The STS score was 1.13 ± 0.48%.

**Table 1:** Baseline characteristics

Variable	Patients (n = 9)
Age, years	50.89 ± 5.30
Male, %	66.67
BMI (kg/m^2^)	23.13 ± 2.09
NYHA functional class, %	
II	55.56
III	44.44
Ejection fraction, %	60.89 ± 8.95
Surgery indication, %	
Severe AS	55.56
Severe AR	44.44
Bicuspid aortic valve, %	66.67
Cardiovascular risk factor, %	
Hypertension	11.11
Diabetes mellitus	0
Active smoking	0
STS predicted mortality, %	1.13 ± 0.48

Operative data are listed in Table 2. Seven (77.78%) patients underwent mechanical valve replacement, and the others (22.22%) successfully received bioprosthetic valve replacement. The prosthetic valve size varies from #17 to #25. Only two patients (22.22%) had concomitant procedures—the aortic root enlargement procedure. No patient underwent conversion to sternotomy. Overall, the mean CPB time was 137.56 ± 27.99 min, and the aortic cross-clamp (ACC) time was 95.33 ± 17.96 min.

**Table 2:** Operative details

Variable	Patients (n = 9)
CPB, min	137.56 ± 27.99
ACC, min	95.33 ± 17.96
Prosthetic valve, %	
Mechanical	77.78
Bioprosthetic	22.22
Prosthetic valve size	
#17	1 (11.11%)
#21	4 (44.44%)
#23	1 (11.11%)
#25	3 (33.33%)
Convert to sternotomy, %	0
Ventilation time, hours	6.13 (4.43, 19.99)
Ventilation time < 24 h, %	88.89
ICU stay, days	2.68 ± 1.30
Blood transfusion, unit	4 (4, 4)
Chest tube drainage, ml	447.67 ± 233.30
In-hospital mortality, %	0
Complication	
Reoperation for bleeding, %	0
Stroke, %	0
Renal failure, %	0
New-onset atrial fibrillation, %	0
Third-degree AV block, %	0

The surgery survival rate was 100%. In both AS and AR patients, the implanted aortic valve works in a good state. Two patients had mild (< 2 cm^2^) perivalvular leakage. The postoperative recovery time was short—the ventilation time was 6.13 (4.43, 19.99) hours; 8 (88.89%) patients extubated within 24 h; and the length of ICU stay was 2.68 ± 1.30 days. The mortality and complication rates were 0%. Only two patients received a four-unit blood transfusion due to massive early postoperative drainage.

All patients were followed up for 1–9 months (median 4 months) by telephone and clinical visits. There was no mortality, vascular complications, valve dysfunction, or new-onset valve diseases during the follow-up. Two patients with perivalvular leakage were kept in the mild degree. Both symptoms and quality of life improved over the follow‐up time.

## Discussion

Since Carpentier first reported minimally invasive mitral valve surgery, thoracoscopy has been widely used in atrioventricular valve surgery, which has also been demonstrated to have favorable results compared to conventional surgery [7, 8]. However, studies on minimally invasive aortic valve surgery are limited, of which most used small incisions and were assisted by endoscopy. Such an incision still has a large wound, so endoscopy is more of an illumination source. In addition, partial sternotomy leads to considerable blood loss and postoperative pain, and even an intercostal incision with a rib spreader may lead to rib fracture or intercostal neural injury. Only a few cases of totally thoracoscopic aortic valve replacement have been reported [9–12]. In 2014, Vola et al. [9] first reported two cases with two working ports positioned in the second (20 mm) and third (15 mm) right intercostal spaces using a 3f Enable sutureless valve. Hinna et al. [11] performed totally thoracoscopic aortic valve replacement through four ports, that is, a 20–30 mm working port in the second right intercostal space and three 5 mm ports in the first, second, and fourth intercostal spaces. Tokoro et al. [12] adopted a surgical route that was the same as that of endoscopic mitral valve surgery on the right anterior thoracotomy to perform totally endoscopic aortic valve replacement. To our knowledge, this is the first report of TTAVR. In our center, we also applied a three-port method to perform totally endoscopic aortic valve replacement and placed the working port in the right third intercostal space on the mid-clavicular line, the assist port in the third intercostal space between the anterior and median axillary line, and the thoracoscopic port in the fourth intercostal space parallel to the assist port. The three-port method has advantages in managing multivalve disease. We drew on previous experience of the difference between two-port and three-port thoracoscopic mitral valve replacement, and we prefer the two-port method for isolated aortic valve replacement (AVR) [13]. The working port in the third intercostal space decreased the distance and angle between the aortic valve and surgical instrumentation, making it easier to suture the aorta incision and knot the valve with a knot pusher. Endoscopy had good quality in the exposure of the noncoronary cusp and was beneficial to avoid atrioventricular block. In addition, endoscopy accessing the thoracic cavity horizontally left more operation spaces and avoided instrument collision.

In this study, a TTAVR was safely performed for selected patients with pure aortic valve disease with no mortality and a durable prosthetic valve during early follow-up. Postoperative cerebrovascular accidents, low output syndrome, and atrial fibrillation were absent, which was in line with Tokoro’s results [12]. Patient recovery was smooth. The lengths of ICU stay and mechanical ventilation were short. The results of blood transfusion, chest tube drainage, and renal failure were also satisfactory. These factors are significant factors for a patient’s fast recovery. At the present stage, TTAVR aims to obtain good outcomes that are comparable to those of SAVR. After that, the performance of such a minimally invasive procedure can be promoted in a larger population, including high-risk, emergency patients. As aortic valve repair could be an attractive alternative to AVR and provides better durability and excellent hemodynamic outcomes in experienced centers [14, 15], the approach and technique in TTAVR might be further transferred to thoracoscopic aortic valve repair.

TTAVR is technically demanding. Even for experienced surgeons, there is a learning curve. Our surgeon, with an annual volume of approximate 250 cases of thoracoscopic surgeries, first had adequate proficiency in the median thoracotomy for valve intervention, then attempted to perform anterior right thoracotomy (the second or the third right intercostal space as access) with and without thoracoscopy assistance for AVR, and finally moved the incision toward the third intercostal distant from the sternum. In the early period of TTAVR, we selected patients with AR instead of AS, so decalcification was not needed, and we could master TTAVR within a limited operating space. In addition, AR is usually combined with a large annule, making it simple to choose valve size and avoid root enlargement procedures. TTAVR for AS is more time spending. We spent an average of 140.3, 99.7 min at CPB, ACC in the first three AS cases. As the learning curve progressed, the overall CPB and ACC times of TTAVR were close to those of totally thoracoscopic mitral valve replacement in our center [13]. For concomitant aortic root enlargement, we controlled the whole CPB and ACC time to approximately 160 and 115 min, respectively. The surgical time might be further reduced with further experience. Besides, with the progression in the new generation of sutureless valves, the lengths of CPB and ACC will also be further shortened. Vola et al. performed TTAVR using a 3f Enable valve and Perceval, presenting faster CPB and ACC times (3f Enable valve: 92 min, 64 min; Perceval: 116 min, 80 min, respectively) [10, 16]. A rapid deployment aortic valve (i.e., Edwards Intuity Elite) applied in a high-volume anterior right thoracotomy center also had CPB (114 min vs. 102 min) and ACC (80 min vs. 76 min) times that approached those in the STS Database for conventional SAVR [17, 18]. Therefore, sutureless and rapid deployment of aortic valves are recommended for isolated AVR, considering some complex cases (such as aged patients and delicate aortic walls) that might benefit from less aortic manipulation and less surgical time [19]. Regardless of the speed and duration of the procedure, the choice of prosthetic valve has a more robust association with patient selection. Cresce et al. [20] reported the largest series of minimally invasive endoscopic AVR covering stented bioprostheses, rapid deployment valves, and sutureless valves. All types of valves are feasible. In their experience, a sutureless valve is more suitable for older patients with a small aortic annulus, while a rapid deployment valve is preferred in younger patients with a small anulus and leftward ascending aorta. Early outcomes of sutureless valves are satisfactory, but further studies for long-term durability, hemodynamic results and complications are required [21].

TTAVR is now limited to a small number of heart centers. In addition to a lengthy learning curve, another reason is the rapid development of transcatheter aortic valve replacement (TAVR). Since the first case of TAVR in 2002, over 700 000 patients have been treated with TAVR worldwide. For aortic valve disease, the role of TAVR has been transferred from “an escape strategy for high-risk patients” to “a safe and considerable option for low-risk patients” [22, 23]. Ghoreishi and his colleague reported the largest dataset of less-invasive AVR and full sternotomy AVR comparison, showing that less-invasive AVR had an extremely low mortality (1%) and fewer complications, such as renal failure and bleeding, in treating low-risk AS patients [24]. The excellent outcomes made less-invasive AVR serve as a benchmark for comparisons between SAVR and TAVR. In our study, TTAVR, as one of the minimally invasive aortic surgeries, performed well not only in AS but also in AR. TTAVR might be able to comparable to TAVR, and it is expected to become a new benchmark. First, TTAVR can remove the diseased aortic valve and surrounding calcifications. In addition, TTAVR provides more size options of the prosthesis, especially for patients with a small aortic annulus size so that they can be matched with the appropriate small size rather than being implanted with the smallest TAVR prosthesis that is too large for them. An additional aortic root enlargement would also ensure that patients have precise sizing of the prosthesis. Based on these two advantages, TTAVR might have less permanent pacemaker implantation and perivalvular leakage than TAVR. On the other hand, lower-risk patients tend to be young or middle-aged patients. TTAVR allows these patients to receive a mechanical valve implant with better durability for their life. We observed no postoperative kidney injury in the TTAVR patients, and one of the reasons for this was that they did not have contrast injections, so TTAVR might be suitable for patients with chronic kidney injury. In some countries (i.e., China), the cost of TAVR valves is still significantly more expensive than that of the available commercial prostheses, making TTAVR potentially cost‐beneficial.

## Limitations

This was a single-center, preliminary study based on a limited number of patients with a short duration and follow‐up. Control group or propensity matching was absent despite the retrospective nature of the study. Future studies about patient selection, procedure expanding indication (e.g., for patients combining aortic root surgery), and long-term follow-up data are awaited.

## Conclusion

In conclusion, TTAVR performed by experienced operators is a safe and effective surgical treatment option for carefully selected patients with pure aortic valve diseases. In the era of catheter-based interventional therapy, TTAVR has advantages and is worthy of widespread adoption. Clinical studies with larger sample sizes and longer follow-up are needed.

## Supplementary Information

**Additional file 1: Video 1.** Concomitant procedure: two-port thoracoscopic aortic valve replacement and aortic root enlargement.
